# Therapeutic Applications of Pretargeting

**DOI:** 10.3390/pharmaceutics11090434

**Published:** 2019-09-01

**Authors:** Marjolein Verhoeven, Yann Seimbille, Simone U. Dalm

**Affiliations:** Department of Radiology and Nuclear Medicine, Erasmus MC, Dr. Molewaterplein 40, 3015 GD Rotterdam, The Netherlands

**Keywords:** pretargeting, radioimmunotherapy, targeted radionuclide therapy, cancer, streptavidin, biotin, bispecific antibody, oligonucleotides, click chemistry, nanoparticles

## Abstract

Targeted therapies, such as radioimmunotherapy (RIT), present a promising treatment option for the eradication of tumor lesions. RIT has shown promising results especially for hematologic malignancies, but the therapeutic efficacy is limited by unfavorable tumor-to-background ratios resulting in high radiotoxicity. Pretargeting strategies can play an important role in addressing the high toxicity profile of RIT. Key to pretargeting is the concept of decoupling the targeting vehicle from the cytotoxic agent and administrating them separately. Studies have shown that this approach has the ability to enhance the therapeutic index as it can reduce side effects caused by off-target irradiation and thereby increase curative effects due to higher tolerated doses. Pretargeted RIT (PRIT) has been explored for imaging and treatment of different cancer types over the years. This review will give an overview of the various targeted therapies in which pretargeting has been applied, discussing PRIT with alpha- and beta-emitters and as part of combination therapy, plus its use in drug delivery systems.

## 1. Introduction

Within nuclear medicine, target mediated radionuclide interventions have emerged as powerful tools for noninvasive molecular imaging and therapy of cancer. Antibodies have been established as potent vectors for delivery of radioactivity because of their high affinity and specificity towards target antigens. A primary concern of the use of radiolabeled antibodies is their slow pharmacokinetics, i.e., a low tumor penetration and low excretion rate [1]. Due to the long residence time of antibodies in the bloodstream, it takes a long time to achieve adequate tumor-to-background ratios after radiotracer injection. For this reason, the use of long-lived radionuclides is required. Besides the dose to the tumor, this results in high effective radiation doses to healthy tissue, in particular, the bone marrow. Clinical complications can occur as a consequence of healthy tissue irradiation, especially when repeated imaging procedures or high therapeutic doses are necessary. Over the past decades, new targeting strategies have been developed to overcome the pharmacokinetic drawbacks caused by the large size of antibodies. One proposed strategy is the use of antibody fragments or engineered antibody formats to enhance tissue penetration. Although this attempt can be successful, it was also observed that a decrease in size is often associated with a lower binding affinity which leads to reduced radioactivity uptake in the tumor [2].

**Figure 1:**
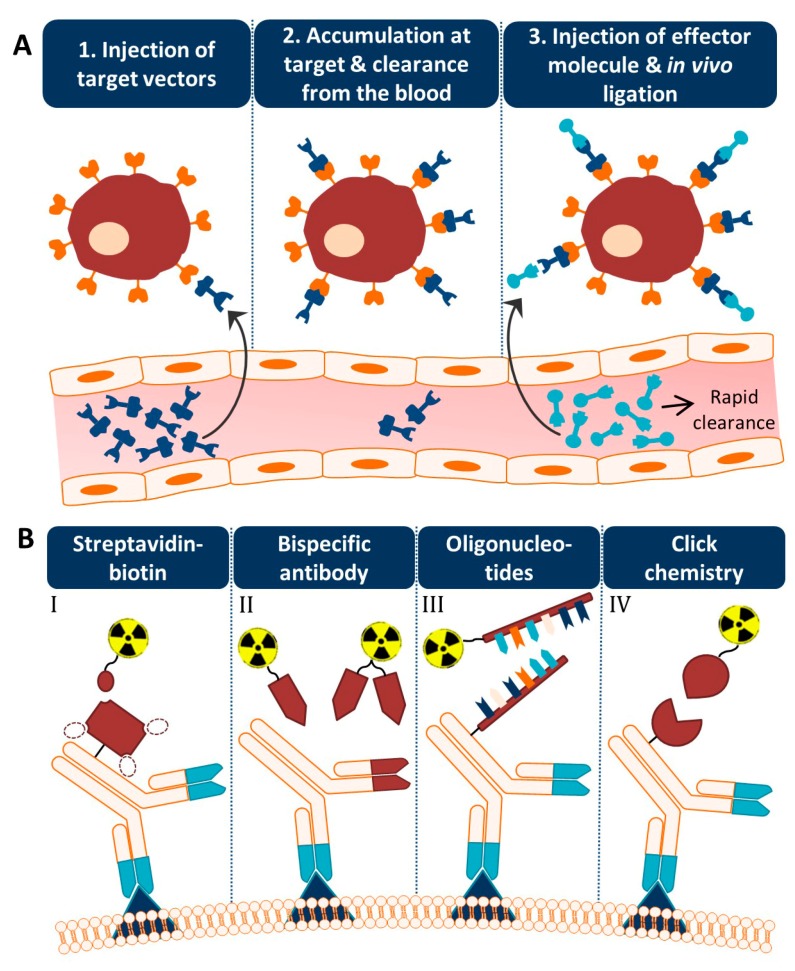
The pretargeting concept. (**A**) Different steps of the pretargeting approach. The targeting vector consists of a binding domain, a linker, and a pretargeting moiety (dark blue) and the small molecule of a complementary pretargeting moiety, a linker, and an effector (e.g., a radionuclide) (light blue). (**B**) The four most commonly applied pretargeting methods are illustrated with an antibody as target vector and a radionuclide as effector. I) The streptavidin–biotin approach. The dotted circles represent the other three biotin binding sites of (strept)avidin. II) Pretargeting using bispecific antibodies with a binding site for a radiolabeled hapten. Two small molecules are shown with on the left a monovalent hapten and on the right a bivalent hapten that could bridge two antibodies (i.e., affinity enhancement system). III) Pretargeting based on oligonucleotide hybridization. The backbone can for example exist of morpholine rings (i.e., MORF/cMORF pretargeting). IV) The click chemistry technology. A covalent bond-forming approach between, for example, *trans*-cyclooctene and tetrazine.

An alternative approach is based on separating the antibody from the radionuclide and letting the two agents combine in vivo [3,4]. This so-called pretargeting concept basically consists of three steps (Figure 1A). In step 1, the targeting vector designed to bind both the target antigen and a radiolabeled small molecule is injected. Once accumulated at the target site and largely cleared from the blood (step 2), a complementary radiolabeled small molecule is administered (step 3). Upon encountering the targeting vector, ligation will take place between the two molecules which leads to the in vivo formation of the radioimmunoconjugate. Due to the small size of the secondary agent, it possesses favorable pharmacokinetic properties, hence the remaining radiolabeled small molecules are rapidly excreted. Occasionally, an extra step with a clearing agent is introduced to remove the unbound targeting vector from the circulation before the injection of the radiolabeled small molecule. The respective clearing agent roughly consists of a binding motif for the pretargeting moiety and a carbohydrate with high affinity for the liver. After injection of the clearing agent, the molecule will rapidly bind the targeting vector, and the newly formed complex will be excreted via hepatic catabolism. The pretargeting strategy thus allows administration of the radiolabeled substance at optimal tumor-to-non tumor (T:NT) ratios, thereby significantly lowering healthy tissue irradiation.

The success of this method is fully dependent on the formation of strong chemical interactions between the complementary reactive groups coupled to the targeting vector and the radiolabeled small molecule under the physiological conditions present in the body. Several bioconjugation techniques with established high binding constants and fast binding kinetics have been studied for pretargeting purposes. Four pretargeting methods are commonly applied (Figure 1B). The avidin–biotin system is based on the strongest noncovalent biological interaction known between biotin and avidin or streptavidin (SA), which possesses four binding sites for radiolabeled biotin molecules [5]. Roughly in the same period, bispecific antibodies with one arm directed against the tumor antigen and one against a radiolabeled hapten (e.g., indium-labeled diethylenetriaminepentaacetic acid (In-DTPA) and histidine-succinyl-glycine (HSG) peptide) were developed [6]. Due to low tumor activity uptake, the use of a bivalent hapten was suggested so that the bispecific antibodies could be crosslinked at the cell surface resulting in binding with enhanced affinity. For this reason, this method is known as the affinity enhancement system [7]. A few years later, a pretargeting method based on the hybridization of an oligonucleotide conjugated to an antibody and a radiolabeled complementary oligonucleotide was generated [8]. In order to enhance the in vivo stability of the oligonucleotides, Liu et al. introduced the use of morpholino backbones (i.e., MORF/cMORF pretargeting) [9]. Bioorthogonal click chemistry was most recently considered as an effective pretargeting strategy, because it exploits pairs of functional groups that rapidly, selectively, and covalently bind in biological systems. One of the proposed click chemistry techniques is the inverse electron demand Diels–Alder (IEDDA) cycloaddition of trans-cyclooctene (TCO) and tetrazine (Tz) which was first introduced in 2010 by Rossin et al. for pretargeted tumor imaging [10]. The mechanisms, advantages, and limitations of each bioconjugation methodology for pretargeting purposes have been extensively discussed in previous reviews [11,12,13,14].

Originally, the use of pretargeting was evaluated for nuclear imaging to improve both contrast and safety. Moreover, it enables the use of radionuclides with shorter half-lives which were otherwise not compatible with the long circulation time of antibodies. However, pretargeting is conjointly very interesting for therapy. It has the ability to enhance the therapeutic index by reducing side effects caused by off-target irradiation and thereby increase curative effects due to higher tolerated doses. Over time, the scope of pretargeting had broadened even further, and next to radionuclide imaging and therapy, this strategy has also been studied for application of other anticancer treatments such as chemotherapeutics.

In this paper, we will provide an overview of the various therapies in which pretargeting strategies have been applied over the years and how it has emerged for each target and purpose. Furthermore, we will discuss novel applications of pretargeting, specifically alpha-particle therapy, combination therapy, and drug delivery.

## 2. Pretargeted Radioimmunotherapy with Beta-Emitting Radionuclides

Treatment using tumor targeting antibodies labeled with cytotoxic radionuclides is called radioimmunotherapy (RIT). RIT is an interesting treatment option for tumor lesions that cannot be surgically removed or easily eradicated by external beam radiation therapy. Since RIT is often associated with high toxicity profiles, the pretargeting strategy has been investigated to improve RIT for both hematologic and solid cancers.

### 2.1. Hematologic Cancers

Although RIT is clinically proven to have a significant antitumor effect in radiosensitive hematologic malignancies such as non-Hodgkin lymphoma (NHL) [15,16,17], the therapeutic efficacy is still limited by the maximum dose that can be safely administered. The bone marrow is usually the dose-limiting organ. Therefore, the pretargeting strategy has been studied for safe and effective RIT directed against various hematological tumor antigens, including CD20, CD45, and CD38.

#### 2.1.1. CD20 Antigen

Since CD20 is highly expressed on lymphomas, CD20-directed RIT has been studied for treatment of this cancer type. Radiolabeled anti-CD20 antibodies have shown promising results in patients with relapsed or refractory B-cell lymphoma. However, the majority of patients presented recurrent disease after treatment, indicating the need for higher tumor doses. To increase therapeutic efficacy while keeping toxicity levels low, pretargeted RIT (PRIT) targeting CD20 antigens has been evaluated in preclinical and clinical studies. In a preclinical study, Press et al. compared RIT and PRIT using yttrium-90 with respect to dose and toxicity in mice bearing human lymphoma xenografts [18]. The authors demonstrated that the RIT doses required for significant tumor responses were associated with lethal toxicity in 100% of the animals, while PRIT with a tolerable two-fold higher dose cured 89% of mice. A comparative study performed by Subbiah et al. produced similar results whilst even showing a cure rate of 100% with PRIT [19]. A clinical phase I/II study with CD20-directed PRIT was published by Weiden et al. in 2001 [20,21]. In this study, performed in relapsed NHL patients, pretargeting also resulted in superior tumor-to-whole body dose ratios when compared to other studies using conventional RIT. Moreover, tumor responses were encouraging as tumors regressed in 6/7 patients treated with PRIT.

In the above studies, pretargeting was performed via the avidin–biotin system. Although results were positive when compared to RIT, patients developed an immune response to SA after treatment. To overcome this problem, a different pretargeting method making use of bispecific antibodies (bsAbs) was explored for CD20 targeting. Comparison of directly labeled anti-CD20 IgG vs. an anti-CD20 × anti-HSG bsAb pretargeting system both labeled with yttrium-90 showed a therapeutic advantage of the latter either when given as a single injection or as fractionated doses [22]. The therapeutic index was even better when a recombinant bsAb was used instead of a chemically conjugated bsAb. Specifically, a 2.6-fold increased tumor uptake and >45-fold improved tumor-to-blood (T:B) ratio were observed [23].

In a second attempt to overcome immunogenicity, engineered fusion proteins (FPs) were applied for CD20-directed PRIT. FPs consist of single or multiple single chain variable fragments (scFv) directed against a specific tumor antigen, that are coupled to SA (scFvSA). Initial preclinical proof-of-concept of such a construct in a PRIT setting was advanced by Schultz et al. who designed and successfully tested an FP based on the CD20-directed antibody B9: a tetravalent scFvSA (scFv_4_SA) [24]. This genetically engineered FP labeled with yttrium-90 was also successfully applied in a clinical pilot study in a NHL patient [25]. Additionally, a phase I trial was performed using this same FP for pretargeting [26]. This study resulted in a tumor-to-whole body dose ratio of 49:1, significantly higher than the reported 38:1 ratio for the SA-conjugated anti-CD20 antibody in the clinical study of Weiden et al. [21]. A direct comparison of a SA-conjugated anti-CD20 antibody 1F5 and a FP for pretargeting based on the same antibody in human lymphoma bearing mice revealed similar tumor uptakes. However, better T:B ratios were obtained with the FP than the antibody conjugate (>65:1 vs. <7:1) [27]. In another study by Green et al., favorable biodistribution of a FP based on the CD20-directed antibody 2H7 was confirmed [28]. Moreover, the authors demonstrated that the pretargeting strategy using this FP and yttrium-90-labeled biotin was highly effective, since all human lymphoma-bearing animals were cured and considerably less myelosuppression was observed.

Alternatively, mutant SA and bis-biotin carriers were used to evade SA immunogenicity and interference of endogenous biotin, which is also a complication of the avidin–biotin system [29]. The application of engineered mutant SA FPs and yttrium-90-labeled bis-biotin reagents enhanced the antitumor efficacy. Mice bearing human lymphoma xenografts treated with the most potent mutant SA FP determined by this study and wild-type SA FP had average tumor volumes of 237 ± 66 mm^3^ and 1129 ± 322 mm^3^ after 11 days, respectively [30]. Frost et al. showed that the therapeutic efficacy is also influenced by the choice of radionuclide. Comparison of lutetium-177 and yttrium-90 for anti-CD20 PRIT showed that a more than two-fold higher absorbed radiation dose could be delivered to a B-cell neoplasm when pretargeting was performed with yttrium-90 instead of lutetium-177 [31].

#### 2.1.2. Other Hematological Tumor Targets

B-cell lymphoma 1 protein (BCL1)-directed PRIT was explored for mantle cell lymphomas as approximately 35–66% of this lymphoma subtype expresses BCL1 [32]. In an initial preclinical study, the use of a pretargeting system based on the anti-BCL1 × anti-HSG bsAb and a complementary iodine-131 bivalent hapten resulted in survival of 14/16 mice without severe toxicity, while directly labeled antibodies induced death from acute toxicity in 3/16 mice [33]. This again demonstrates the potential of PRIT to reduce toxicity. Next to CD20, CD22 and the human leukocyte antigen-DR have also been investigated as targets in clinical RIT studies. For all antigens, it holds that PRIT is superior over RIT in terms of biodistribution [34]. The success of the treatment, however, depends on the antigenic expression which is different in each lymphoma cell line. Interestingly, no synergistic or additive benefit was observed when a combination therapy of all three antibodies was given. In a pediatric anaplastic large cell lymphoma patient, immunohistochemistry indicated the presence of tenascin and using this target for PRIT with yttrium-90 resulted in a remarkable complete remission as the patient was resistant to all other treatments [35].

In patients with acute myeloid leukemia (AML), RIT directed against the CD45 antigen has shown encouraging results, making it an interesting target for this patient group. Lin et al. developed and evaluated an anti-CD45 scFv_4_SA for PRIT of AML to overcome the limitations of RIT regarding high toxicity and subsequent limited cure rates [36]. The preclinical study performed in mice bearing CD45 positive human lymphoma xenografts resulted in 100% survival when treated with high dose PRIT using yttrium-90. One major issue in preclinical anti-CD45 PRIT research concerns the use of xenograft mouse models. Mouse hematopoietic cells lack CD45 expression, while in human subjects, CD45 is expressed on tumors cells and hematopoietic cells. Therefore, Pagel et al. used a syngeneic murine leukemia model in which normal myeloid, lymphoid, and reticuloendothelial tissues expressed the CD45 antigen [37]. Therapy studies demonstrated high T:NT ratios and significantly prolonged survival compared to conventional RIT in this model [38]. The therapeutic advantage of anti-CD45 PRIT was also shown in nonhuman primates [39], again suggesting that applying a pretargeting strategy is superior to conventional RIT and even allows intensification of treatment schedules. Since CD45 is stably expressed with high density on the majority of hematopoietic cells, CD45-directed therapy can provide an alternative for CD20 negative lymphoma patients. A comparison was made between anti-CD45 and anti-CD20 antibodies for PRIT in mice bearing human lymphoma xenografts that were CD20 and CD45 positive [40]. Both anti-CD45 and anti-CD20 PRIT were effective, but anti-CD45 antibodies resulted in the delivery of two- to four-fold more radiation to the tumor than the anti-CD20 antibodies. It is likely due to the superior retention of this antibody on the cell surface.

In contrast to NHL and AML, very little attention has been paid to the role of RIT and even PRIT in multiple myeloma (MM). The CD38 transmembrane glycoprotein retains at high density and uniform expression on MM cells and is minimally expressed on normal hematopoietic cells. In a preclinical study, anti-CD38-SA PRIT with yttrium-90 reached long-term myeloma-free survival in all animals bearing human MM tumor xenografts and T:B ratios of 638:1 compared to <1:1 for conventional RIT [41]. A direct comparison was made between two pretargeting strategies directed against CD38, the streptavidin–biotin based approach and a bispecific FP developed to eliminate SA related immunogenicity [42]. The results of this preclinical study showed that at the highest radiation dose tested, cure rates for the two approaches were similar, while at lower doses the bispecific FP outperformed CD38-SA.

### 2.2. Solid Cancers

In contrast to treatment of hematologic malignancies, RIT has failed to be effective for solid tumors because of their lower radiosensitivity [43]. Moreover, in advanced stages where tumors are bulkier and less vascularized, tumors are less accessible to antibodies resulting in limited tumor radioactivity uptake and thus low T:NT ratios. These factors contribute to suboptimal therapeutic indexes since high doses need to be administered to obtain a therapeutic effect. Furthermore, the bone marrow is, similar to hematologic malignancies, also the dose-limiting organ for RIT in solid tumors. Introduction of pretargeting strategies can provide a safe and effective approach for RIT of solid tumors. PRIT targeted against several tumor antigens, including the carcinoembryonic antigen (CEA) and the tumor-associated glycoprotein 72 (TAG-72), have been studied for treatment of solid tumors and will be discussed below.

#### 2.2.1. Carcinoembryonic Antigen (CEA)

CEA is one of the most widely used tumor markers. CEA is highly expressed in colorectal carcinoma (CRC), medullary thyroid carcinoma (MTC), and small cell lung cancer (SCLC), with the first two being the subject of many preclinical studies. A comparative study of the affinity enhancement system (AES) with a murine anti-CEA × anti-DTPA-In bsAb and conventional RIT in a LS174T human CRC animal model demonstrated the superiority of pretargeting [44]. Iodine-131 treatment with the directly labeled antibody resulted in a growth delay of 53 ± 5 days while PRIT resulted in efficient growth inhibition >150 days. Besides, a nearly 10-fold higher dose could be administered safely in terms of both hematologic and nonhematologic toxicity. A direct comparison between this bsAb and a bsAb with a humanized anti-CEA arm both labeled with rhenium-188 showed that the chimeric antibody was cleared more rapidly from the blood because it was recognized by the reticuloendothelial cells from the spleen [45]. However, the authors argued that this recognition mechanism may be unique for the mouse model and will not be observed in clinical settings. Although a faster blood clearance results in less tumor targeting, Yazaki et al. still observed that lutetium-177-based pretargeting reached a much higher T:B ratio than the direct targeting approach, 199:1 vs. 3:1 respectively [46]. Karacay et al. showed that higher doses could be tolerated with PRIT using fully humanized anti-CEA × anti-HSG bsAbs and yttrium-90-labeled haptens [47]. A 2.5-times higher tumor dose directly translated into improved survival with 33% tumor ablation versus 100% tumor viability in the conventional RIT treated animals bearing human CRC tumors. The same cure rate was reported by Gautherot et al. when treating human CRC xenografted mice with PRIT using murine anti-CEA × anti-DTPA-In bsAb and an iodine-131-labeled hapten [48]. The same pretargeting system has also been applied to human MTC xenografted mice and this was compared to RIT using radiolabeled antibody fragments consisting of two antigen-binding portions linked together (F(ab’)_2_) [49]. Both methods resulted in high tumor uptake values of 8.0 and 7.39%ID/g at 24 h, respectively. However, a higher T:B ratio of 37:1 vs. 1.8:1 was observed for PRIT vs. RIT with iodine-125, respectively. Additionally, Kraeber-Bodéré et al. showed less toxicity for the PRIT treatment group with iodine-131 [50].

CEA is the most extensively studied solid tumor target for PRIT purposes and a lot of research has been performed to optimize conditions for anti-CEA PRIT. This includes preclinical studies regarding the bsAb/hapten molar ratio injected and time interval between injections. The pretargeting efficacy is an interplay between both pretargeting parameters. Gautherot et al. demonstrated that increasing the delay between the injection of the anti-CEA × anti-DTPA-In bsAb and the iodine-131-labeled bivalent hapten from 15 h to 20 h significantly reduced myelotoxicity caused by circulating activity [48]. Sharkey et al. showed that an anti-CEA × anti-HSG bsAb/indium-111-labeled hapten injection ratio of 50:1 combined with a 48 h delay resulted in a 1.6-fold higher tumor uptake than a 10:1/24 h combination [51]. The authors demonstrated that increasing the bsAb dose should be accompanied by lengthening the time interval to maintain adequate T:B ratios. Alternatively, Mirallié et al. tested an avidin chase to remove excess biotinylated bsAbs from the circulation before the hapten injection and showed 3.5-fold improved T:B ratios [52].

**Figure 2:**
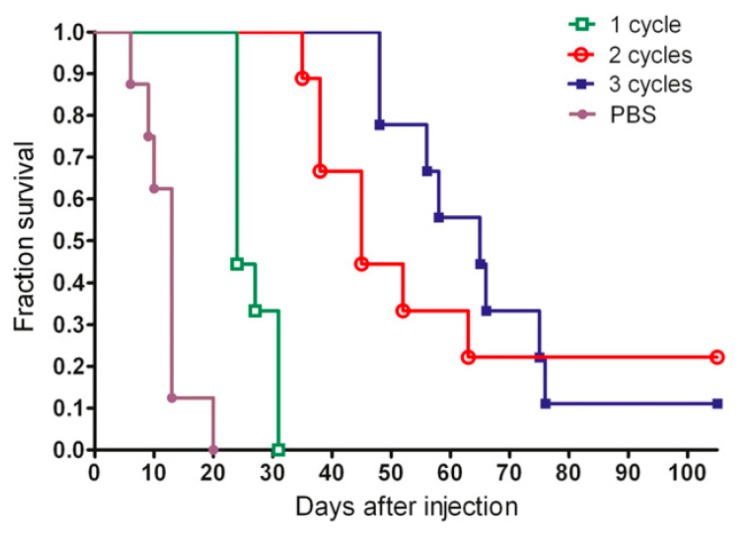
Representative example of the use of multiple pretargeted radioimmunotherapy (PRIT) treatment cycles. Survival curve of mice bearing human CRC tumors divided in groups receiving either 1, 2, or 3 cycles of PRIT with anti-CEA × anti-HSG bsAbs and lutetium-177-labeled DOTA-di-HSG peptide or PBS. This research was originally published in JNM. Schoffelen et al. Pretargeted 177Lu Radioimmunotherapy of Carcinoembryonic Antigen–Expressing Human Colonic Tumors in Mice. *J Nucl Med*. **2010**, *51*, 1780–1787. © SNMMI. [55].

Furthermore, studies have been performed to investigate whether the therapeutic efficacy of anti-CEA PRIT can further be optimized by performing repeated treatments to reach curative doses. The success of this strategy partly depends on the CEA expression levels after a single cycle of PRIT. Immunohistological analysis showed that the CEA status remained unchanged in MTC tumors, but that CRC lesions presented a 58% loss of CEA membrane expression [53]. The first study was performed by Kraeber-Bodéré et al. who confirmed that repeated treatments of PRIT based on the AES with murine anti-CEA × anti-DTPA-In bsAbs and iodine-131-labeled haptens in mice xenografted with human MTC led to high antitumor efficiency without a significant increase in toxicity [54]. Generally, a longer tumor response was achieved with the remarkable exception of two complete responses after two therapy cycles. Successive cycles of anti-CEA × anti-HSG bsAb and lutetium-177-labeled hapten based PRIT also effectively delayed the growth of human CRC tumors and enhanced survival from 13 to 65 days for mice treated with three cycles vs. one cycle of PRIT [55]. Interestingly, three cycles did not outperform two cycles (Figure 2), probably due to decreased tumor uptake during the third cycle which might be caused by a lower CEA expression as previously reported.

Not only can pretargeting parameters or the treatment schedule influence the therapeutic index, but the pretargeting method also can. The chelator and the radionuclide of choice can play an important role. In a LS174T human CRC mouse model, bivalent haptens labeled with various radionuclides (i.e., indium-111, yttrium-90 and iodine-131) against the anti-CEA × anti-DTPA-In BsF(ab’)_2_ proved to have a higher targeting efficiency than the monovalent hapten [56]. Gestin et al. pointed out that the greater range of rhenium-188 could be beneficial over iodine-131 for radiolabeling of haptens in the treatment of larger CRC tumors with a diameter up to 1 cm [57]. Most preclinical anti-CEA PRIT studies have been performed with the AES, but few are based on the avidin–biotin system. For this approach, higher T:NT ratios were obtained when biotin was coupled to 1,4,7,10-tetraazacyclododecane-1,4,7,10-tetraacetic acid (DOTA) instead of DTPA to complex yttrium-90 [58]. The MORF/cMORF pretargeting system also led to promising results as anti-CEA PRIT with rhenium-188 ceased tumor growth after 1 day, contrary to continued growth in the control group [59].

A lot of effort has been made to translate anti-CEA PRIT to the clinic. Already in 1996, Bardies et al. performed a feasibility study using the pretargeting approach with murine anti-CEA × anti-DTPA-In bsAbs and iodine-131-labeled bivalent haptens in 10 patients with MTC and SCLC [60]. Interestingly, a higher uptake was detected in recurrences of MTC than of SCLC, even though CEA expression levels of primary tumor biopsies (used as an inclusion criterion for the study) were similar. This could indicate that CEA expression levels of the primary tumors are not predictive for expression levels in recurrent disease. Hence, imaging beforehand could help to select eligible patients for PRIT. Using the same pretargeting system, Chatal et al. demonstrated long-term efficacy in terms of survival benefit in 29 patients with rapidly progressive metastatic MTC [61]. This study provided the first proof of effective RIT in solid tumor patients with confirmed metastatic disease; however, here, a pretargeting strategy was applied and essential for the observed success. It was determined that PRIT treated patients with a serum calcitonin doubling time (Ct DT), a prognostic biomarker for survival in MTC, of less than 2 years (high-risk group) had a significantly longer survival than the corresponding untreated control group; 110 vs. 61 months, respectively.

Clinical translation can be hampered by the development of a significant human anti-mouse antibody response. Ideally, such a response should be avoided and for this reason a phase I optimization trial was initiated using a chimeric anti-CEA × anti-DTPA-In bsAb and iodine-131-labeled bivalent hapten [62]. A bsAb dose of 40 mg/m^2^ was preferred over 70 mg/m^2^ as it significantly decreased the risk of severe hematologic toxicity. However, it came at the cost of therapeutic efficacy in terms of the disease stabilization rate, 22% versus 64% respectively. Additionally, it was determined that MTC patients possessed a higher risk of hematologic toxicity than patients with other CEA-expressing tumors. This suggests that PRIT dose schedules should be altered for each tumor type. Serious hematologic side effects of anti-CEA PRIT with iodine-131 were also observed in a phase II trial in advanced and progressive MTC patients [63]. However, a high disease control rate of 76% was reached and treatment appeared to be effective in patients with short Ct DTs, suggesting limiting the application of PRIT to the poor prognostic risk group.

Despite the use of chimeric bsAbs, immunogenicity was still present and therefore efforts were made to prepare fully humanized constructs. Pretargeting based on the new generation of humanized, recombinant, trivalent anti-CEA × anti-HSG bsAbs named TF2 was shown to be safe for the treatment of metastatic CRC patients with the lutetium-177-labeled hapten IMP288 [64]. Subsequent dosimetry data based on a pretherapy diagnostic study where the hapten was labeled with indium-111 could accurately predict the maximum tolerated dose in a PRIT situation [65]. The authors improved the dosimetry model and predicted that tumor-to-bone marrow ratios would increase by an average of 25% when using yttrium-90 instead of lutetium-177 for therapy [66]. The new compounds were also utilized in a phase I/II PRIT trial for patients with advanced lung cancer [67]. In this study focusing on optimization, the importance of an imaging session beforehand was once again confirmed since this was shown to be predictive for absorbed doses in therapy. Furthermore, the study determined that a 24 h delay and a peptide dose of 240 nmol/m^2^ were better pretargeting parameters for therapy than a 48 h delay and a 480 nmol/m^2^ dose.

#### 2.2.2. Epithelial Cell Adhesion Molecule

The epithelial cell adhesion molecule (EpCAM) is a 40 kDa epithelial antigen that is expressed on the surface of most human epithelial carcinomas (e.g., lung, colon, and breast). The NR-LU-10 murine monoclonal antibody recognizes this glycoprotein and is therefore the targeting vector of choice. Axworthy et al. demonstrated improved efficacy of the pretargeting strategy using this monoclonal antibody coupled to SA combined with a biotinylated galactosyl-human serum albumin clearing agent in various cancer types, including SCLC, CRC and breast cancer [68]. A single dose of yttrium-90-labeled DOTA-biotin resulted in 28/30 cures in mice bearing human SCLC, CRC, and breast cancer xenografts as compared to 1/30 cures obtained with conventional RIT in these same animal models. The observed therapeutic advances are most likely due to the >20-fold improvement in T:B ratio and increased absolute tumor doses. Similar high cure rates were reached in the study of Goshorn et al., who additionally showed that the use of an EpCAM-targeting FP is preferred over the normal monoclonal antibody [69]. The FP had a two-fold better antigen binding plus a faster blood clearance which in turn reduced blood radioactivity exposure by four times.

The encouraging experimental results initiated a clinical phase I study in 43 patients with adenocarcinomas [70]. In an optimized setting, PRIT with yttrium-90-labeled DOTA-biotin was able to deliver a 63:1 tumor-to-bone marrow absorbed dose ratio, substantially higher than the 6:1 ratio reported for conventional RIT. Coinjection of an indium-111-labeled tracer dose in a 1:50 ratio with the yttrium-90-labeled tracer enabled the accurate estimation of these radiation absorbed doses. The feasibility of the method that involved the quantification of the activity in gamma camera images was previously described by Breitz et al. [71]. Subsequently, a phase II study was performed in 25 patients with metastatic colon cancer [72]. Treatment, however, appeared to be unsatisfactory in terms of efficacy and both hematological and overall toxicity.

As became apparent from the observed toxicity levels, the therapeutic index was negatively affected by the cross-reactivity of NR-LU-10 antibody with naturally expressing EpCAM tissues such as the intestinal epithelium and kidney tubules. This underlines the importance of targeting antigens that are highly expressed in tumor tissue but have no/little expression in healthy organs. In 2003 Lewis et al. published a preclinical study using the NR-LU-10 antibody, yet their goal was to encourage the use of the intermediate half-lived radionuclide copper-64 (half-life = 12.7 h) [73]. The rapid pretargeting approach enabled efficient delivery within ~1 h compared to ~48 h with direct targeting.

#### 2.2.3. TAG-72

In contrast to NR-LU-10, the murine CC49 antibody directed against TAG-72 does not demonstrate cross-reactivity with healthy tissues. TAG-72 is present on a variety of adenocarcinomas, but the utility of PRIT is mainly validated in CRC. PRIT of subcutaneous human CRC xenografts using CC49 coupled to SA and yttrium-90-labeled DOTA-biotin was proven to be safe when administered intraperitoneally (i.p.) [74]. At least nine-fold higher doses were safely administered when compared to the application of directly labeled yttrium-90 antibodies, but only a modest decrease of 30–40% in growth rate was achieved. However, application of a pretargeting approach using FPs and the inclusion of an extra step in which a synthetic clearing agent with multiple *N*-acetyl-galactosamine residues coupled to biotin was introduced, showed significantly improved survival from 37 days for controls to 60 days for animals treated i.p. with lutetium-177-labeled DOTA-biotin [75]. A phase I clinical study in chemoresistant TAG-72 positive metastatic CRC patients with a CC49 FP and yttrium-90-labeled DOTA-biotin presented eight- to 11-fold increased T:NT ratios with an impressive 139:1 tumor-to-bone marrow ratio [76,77]. The enhanced biodistribution was unfortunately accompanied by immunogenicity and thus therapy was restricted to a single cycle.

The idea of using SA-coupled CC49 fusion proteins for PRIT purposes was rejected due to the observed immunogenicity of SA. Therefore, alternative approaches have been invented such as CC49 PRIT by MORF/cMORF pretargeting which resulted in temporary complete responses in 3/5 human CRC xenografted mice [78]. Interestingly, targeting of CC49 was shown to be equally effective as anti-CEA PRIT in a CRC animal model. A follow-up study comparing rhenium-188- with yttrium-90-conjugated cMORF detected similar tumor retention and clearance for both radionuclides. Exploiting the longer half-life of yttrium-90 was considered to be beneficial since it would provide higher T:NT absorbed dose ratios [79].

A comparison between radionuclides for PRIT with CC49 FPs was first carried out in 2004 where analyses of the three radionuclides promethium-149, hollium-166, and lutetium-177 determined that the best therapeutic index belonged to lutetium-177 [80]. This finding was later confirmed by Mohsin et al. who also concluded that lutetium-177 was the optimal choice as it increased the median time to progression from 13 to 50 days based on PRIT with tetravalent CC49 FPs [81]. In addition, five-times higher doses than with conventional RIT could be safely delivered, resulting in more long-term survivors. Rossin et al. used lutetium-177 and implemented this in their pretargeting method based on the IEDDA reaction in another attempt to avoid immunogenicity and hence offering the possibility of repeated treatments [82]. When compared with conventional lutetium-177 RIT, PRIT boosted T:B ratios and allowed for eight-fold higher tumor doses while decreasing toxicity. The potential of pretargeting for radionuclide therapy using antibody fragments, which is often hampered by nephrotoxicity, was shown by van Duijnhoven et al. [83]. A >20-fold higher tumor-to-kidney ratio was obtained with lutetium-177-labeled Tz and TCO functionalized TAG-72-binding diabodies than with directly labeled diabodies.

#### 2.2.4. Tenascin-C

Tenascin-C is an extracellular matrix protein abundantly expressed in the stroma of several solid tumors such as melanoma and malignant glioma. For gliomas, tenascin expression is positively correlated with tumor grade. Accordingly, PRIT with a biotinylated anti-tenascin antibody, followed by avidin injection to provide binding sites for yttrium-90-labeled biotin was performed in 48 patients with high-grade glioma [84]. This phase I/II clinical study demonstrated an objective tumor reduction and disease stabilization accompanied by low toxicity in 25% and 50% of patients, respectively. As a result of the promising therapeutic efficacy, the same pretargeting approach was evaluated in an adjuvant setting; pretargeted therapy was started one month after conventional treatment [85]. Treated glioblastoma patients had a significantly longer survival time than control patients with a median survival of 33.5 versus 8 months, respectively. The same group continued treating numerous glioblastoma patients with recurrent disease after standard treatments. A large scale retrospective study, analyzing data of these 502 glioblastoma patients treated with tenascin-C-directed PRIT, reported a median survival of 19 months with 8% of patients showing tumor mass reduction and 28% presenting stable disease [86]. In accordance with other PRIT studies, high immunogenicity of SA was observed as almost all patients (90%) developed anti-SA antibodies.

Since anti-tenascin monoclonal antibodies have been effective in intratumoral administration of RIT [87], a phase I study was performed to evaluate whether applying a pretargeting approach for locoregional RIT could also be of benefit. In 24 patients with recurrent glioma, therapy was safely administered via a catheter, which was placed in the surgical resection cavity after the second surgical debulking [88]. The response rates were in accordance with the previous phase I/II study. Even higher tumor doses can be delivered when biotin conjugates are resistant to the enzymatic action of biotinidase. Therefore, Urbano et al. designed a new biotin-DOTA conjugate to enhance PRIT efficiency by preventing enzymatic degradation while maintaining high binding affinity for SA [89]. A pilot experiment in metastatic melanoma patients showed favorable kinetics and high tumor uptake.

#### 2.2.5. Other Solid Tumor Targets

Over the past decades, multiple solid tumor targets have been explored. In the early days of pretargeted therapy, when the focus was primarily on demonstrating its potential, experiments were performed using an antibody specific for yttrium-88-labeled DOTA that localized in the tumor via passive diffusion through the leaky vasculature. Goodwin et al. were the first to apply this pretargeting approach for RIT [90]. Supported by the previous development of the DOTA bifunctional chelator, high selective tumor uptake and promising T:B ratios were obtained with PRIT using a large molecular weight polyvalent hapten as clearing agent. A more thorough investigation into the biodistribution and dosimetry of this approach determined high therapeutic ratios. This was particularly caused by low blood and bone marrow doses as a result of the rapid clearance from all organs, and the stable DOTA chelation reducing the amount of free yttrium-90 that could potentially accumulate in bone [91].

To avoid the choice of selecting the appropriate target, administration of a cocktail of three different biotinylated antibodies, i.e., anti-TAG72, anti-CEA, and anti-tenascin, was investigated by Cremonesi et al. [92]. The authors determined that patients with various tumors were able to withstand higher activities using this strategy than with conventional RIT. Besides, the importance of stable chelation was again confirmed as the difference between DTPA (with less stable chelation) and DOTA revealed that unbound yttrium-90 was mainly responsible for observed hematopoietic effects. A different combination of anti-MOv18 directed at the folate receptor α, anti-TAG72, and anti-CEA monoclonal biotinylated antibodies and yttrium-90-labeled DOTA-biotin was well tolerated in 38 advanced ovarian cancer patients when administered either i.p. or i.p. plus intravenously (i.v.) [93]. The fact that in a relatively large percentage of patients tumor reduction or disease stabilization (9% and 32% respectively) occurred was remarkable since all patients were no longer responsive to conventional treatments. This implies that PRIT could function as an effective treatment for end-stage ovarian cancer patients.

The issue of the most advantageous target for PRIT or RIT and in this case specifically for SCLC, was also addressed by Hosono et al. [94]. The authors stated that the neural cell adhesion molecule (NCAM) could serve as a complementary target for SCLC, since CEA is only heavily expressed in <35% of cases. Anti-NCAM × anti-histamine bsAb and radioiodinated bivalent hapten-based PRIT resulted in higher T:NT ratios suggesting a dosimetry benefit for PRIT using bsAbs compared to treatment with directly radiolabeled antibodies. Sato et al. evaluated mesothelin as target antigen, which is overexpressed on mesotheliomas and ovarian cancers [95]. Although a high therapeutic efficacy was observed with 86% more survivors after 110 days than in the untreated mice population, PRIT using tetravalent FPs directed against mesothelin is challenging due to possible cross-reactivity with normal mesothelin-expressing tissues (e.g., pleura, pericardium, and peritoneum). Another study focused on the Lewis^y^ antigen, which is expressed on the cell surface of many different carcinomas [96]. In contrast to RIT, the PRIT approach based on the avidin–biotin system using yttrium-90 resulted in good tumor responses in mice xenografted with a human epidermoid carcinoma cell line [97].

Cheal et al. selected disialoganglioside (GD2) and glycoprotein A33 (GPA33) as targets for PRIT in neuroblastomas and CRC, respectively. PRIT using an anti-GD2 × anti-DOTA scFv bsAb construct and a lutetium-177-labeled hapten resulted in complete tumor responses in 5/5 animals carrying subcutaneous human GD2 positive neuroblastoma xenografts treated with three successive cycles [98]. Similar responses were seen for two-cycle anti-GPA33 PRIT with lutetium-177, but only 2/9 mice bearing human CRC xenografts showed no recurrence after >140 days [99]. After immunohistochemical analysis of the residual tumors, it was concluded that tumor regrowth was likely because of insufficient radiation absorbed doses to the tumor rather than a decrease in target expression. The addition of a third cycle to the fractionated regimen proved to be essential for cure [100]. Van Rij et al. also investigated the potential of multiple cycles of PRIT, but used a trophoblast cell-surface antigen 2 (TROP-2) positive prostate cancer model. They showed no additional benefit of three cycles of PRIT using a lutetium-177-labeled peptide compared to RIT; PRIT was at least as effective with similar hematological toxicity [101]. However, these results are not in line with results generally obtained in preclinical PRIT studies and can be assigned to the decreased tumor affinity of the anti-TROP-2 bsAb used in this study. This clearly indicates the importance of choosing a good vehicle in addition to a suitable target.

**Figure 3:**
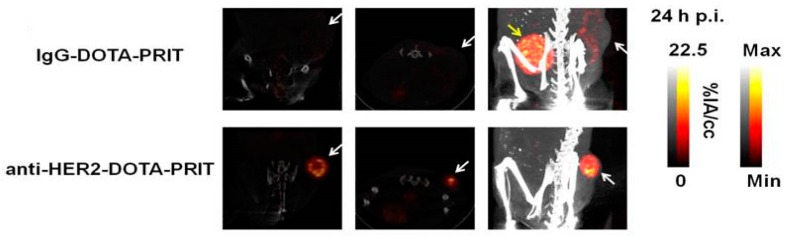
Representative images demonstrating feasibility of PRIT for internalizing targets. SPECT/CT images (from left to right: coronal and transverse slices through center of tumor, maximum intensity projection) of human breast cancer xenografts 24 h postinjection of one cycle of lutetium-177-labeled DOTA-Bn hapten pretargeted with either control IgG-DOTA or anti-HER2 × anti-DOTA bsAb. The white arrow is pointed at the tumor and the yellow arrow at the bladder. The color scale represents the percentage of injected activity per cc. This research was originally published in Theranostics. Cheal et al. Theranostic pretargeted radioimmunotherapy of internalizing solid tumor antigens in human tumor xenografts in mice: Curative treatment of her2-positive breast carcinoma. *Theranostics* **2018**, *8*, 5106–5125. [103].

Traditionally, it was understood that a non-internalizing target provided better accessibility for the secondary agent. This understanding has recently been challenged by studies demonstrating the feasibility of PRIT for internalizing HER2 receptor complexes in breast cancer. Though it was shown by Van Rij et al. that high and fast accumulation can be achieved with internalizing anti-TROP-2 bsAb-antigen complexes [102], the first case of curative and safe fractionated PRIT was reported by Cheal et al. [103]. Anti-HER2 × anti-DOTA-Bn bsAb and lutetium-177-labeled hapten based fractionated 3-cycle PRIT resulted in successful tumor targeting of HER2 positive BT-474 human breast cancer xenografts (Figure 3) and demonstrated successful antitumor efficacy. Another study that focused on anti-HER2 PRIT used an affibody-based peptide nucleic acid pretargeting strategy. Using this pretargeting strategy, anti-HER2 PRIT with lutetium-177 has also been shown to improve survival in mice bearing HER2-expressing human xenografts and has overcome the problems of renal toxicity normally associated with affibodies [104].

The GPA33 and the pancreatic ductal adenocarcinoma (PDAC) biomarker carbohydrate antigen 19.9 (CA19.9) have been explored for bioorthogonal click chemistry-based PRIT. After promising results with this approach during imaging and biodistribution studies, Houghton et al. were the first to perform a longitudinal therapy study [105]. Pretargeted therapy with a TCO-modified anti-CA19.9 antibody and different doses of lutetium-177-labeled Tz illustrated a dose dependent antitumor effect with the highest dose of 1200 mCi resulting in effective tumor regression in 6/8 mice bearing human PDAC xenografts. A dosimetry study by Membreno et al. showed that the effective dose of an IEDDA based anti-huA33 PRIT also with lutetium-177-labeled Tz in human CRC bearing mice was only marginally lower than with conventional RIT (0.054 vs. 0.068 mSv/MBq) [106]. However, the dose delivered to the normally dose-limiting bone marrow was nearly halved, thus allowing treatment intensification of PRIT.

Recent innovations have led to the possibility to create a local tumor target for targeted radionuclide therapy in an intraoperative setting. The injection of avidin into and around the tumor bed prompts ‘avidination’ of the tissue; avidin serves as an artificial receptor. A proof-of-principle study in 11 breast cancer patients confirmed homing of i.v. administered yttrium-90-labeled biotin to avidin in the tumors which resulted in fast and stable uptake of radioactivity [107]. The oxidized form of avidin (avidinOX) possesses a longer tissue residence time than native avidin. In this setting, the use of avidinOX and subsequent therapy with yttrium-90-labeled biotin resulted in eradication of neoplastic breast cancer lesions in mice [108]. Albertoni et al. focused on the application of avidinOX for local treatment of tongue cancer masses, thereby illustrating its potential utility for unresectable tumors [109]. This approach produced significant antitumor activity whilst preserving the integrity and function of healthy tissue.

## 3. Pretargeted Alpha-Particle Therapy

The choice of radionuclide has considerable impact on the effectiveness of targeted radionuclide therapy. The use of alpha particles as the cytotoxic agent could offer significant advantages over beta particles for treatment of metastatic or minimal residual disease because of the short path length in tissues (<100 µm) and high linear energy transfer (~100 keV/µm) of these particles. Beta particles have a lower energy transfer compared to alpha particles, and cause DNA damage mostly via an indirect effect. This is in contrast to the direct DNA damage induced by alpha particles, which is independent of the formation of reactive oxygen species. In contrast to the single strand DNA breaks caused by beta particles, DNA double-strand breaks caused by alpha particles are harder to repair and therefore more frequently result in cell death. Concerning the range, beta particles penetrate more easily through tissue which could be beneficial in case of tumor heterogeneity, but in case of small tumor lesions, a large fraction of their energy is deposited outside the tumor area. For this reason, targeted alpha therapy is considered to achieve a higher therapeutic efficacy for microscopic tumors. Pretargeting strategies compatible with two promising alpha emitters bismuth-213 and astatine-211, possessing short half-lives of 46 min and 7.2 h respectively, were introduced. Park et al. showed that directly labeled antibodies with bismuth-213 were not effective in delivering radioactivity to the tumor because most radioactive decay occurred in the bloodstream [110]. The application of a pretargeting strategy improved RIT using this alpha-emitting radionuclide. The authors demonstrated that alpha PRIT directed against CD20 significantly prolonged survival in mice bearing human lymphoma xenografts.

Alpha PRIT directed against CD25 can play an important role in addressing isolated malignant cells often present in leukemia. This highly localized treatment is especially advantageous for cells critically located in the bone marrow. Besides the benefit of a pretargeting approach, here, the use of short-range alpha-particles emitted by bismuth-213 prevent adverse effects as a result of hematopoietic stem cell irradiation [111,112]. Pagel et al. also showed the potential of bismuth-213 alpha PRIT. In their study, anti-CD45 alpha PRIT proved to be 60% more effective than PRIT with beta-particle irradiation for treatment of AML while minimizing toxicity [113]. Next to leukemia, attention has been paid to the role of alpha PRIT with astatine-211 for intracavity treatment of disseminated ovarian microtumors. Although therapy directed at the membrane transporter SLC34A2 was injected i.p. and thus not influenced by the prolonged circulation time of antibodies, the limiting factor in this case was the slow tumor penetration compared to the radionuclide half-life. In this situation, pretargeting with small molecules was successfully applied to achieve a faster and more homogeneous biodistribution [114,115].

Debate continues about the best choice of alpha-emitting radionuclide for alpha PRIT. Astatine-211 is considered best at delivering high tumor doses to CEA-expressing lesions as deduced from pharmacokinetic modeling [116]. It has become apparent from previous studies that in contrast to the hematologic toxicity of conventional RIT, the main limitation of alpha PRIT is the risk of nephrotoxicity [117,118]. Several alternative strategies have been suggested to reduce nonspecific radioactivity uptake in the kidneys, such as adjusting the charge and size of the small molecule [119], an oral administration route [110], chelate therapy [120], dose fractionation, or the use of longer lived radionuclides to reduce the percentage of decay in the kidneys. Recently, investigators have examined the use of the longer lived alpha-particle emitting radionuclide, actinium-225, for pancreatic ductal adenocarcinoma. However, here, the release of radioactive unbound daughter nuclides with intrinsic off-target kidney affinity might complicate clinical translation [121].

## 4. Combination Therapy

The past decades have seen increasingly rapid advances in the field of combination therapy for cancer management. For instance, chemoradiotherapy is now an established treatment approach. Of particular concern is the increase in toxicity when combining two treatment modalities. Since pretargeted therapeutic strategies have been designed to lower the toxicity of RIT, particularly hematologic toxicity, they are interesting to combine with other anticancer treatments such as myelosuppresive chemotherapeutics, or to serve as adjuvant care. It has been illustrated that the antitumor efficacy of PRIT can be enhanced in a synergistic manner by applying radiosensitizing chemotherapeutic drugs like paclitaxel [122] or gemcitabine [123,124]. The use of PRIT in combination therapy has also been shown to be effective in eradicating residual disease. Either in combination with the alkylating agent temozolomide, microscopic disease outside the radiation field of locoregional PRIT was eliminated [125], or PRIT was used in an adjuvant setting after surgery and radio-chemotherapy [126]. Moreover, administration of immunotherapy directed against the same target after PRIT has been shown to produce an enhanced antitumor response [111,127].

## 5. Novel Applications of Pretargeting: Beyond Antibodies as Targeting Vector

Nanocarriers, such as liposomes and nanoparticles (NPs), have emerged as promising vehicles for cancer-targeted drug delivery. Encapsulating therapeutic molecules facilitates the use of hydrophobic anticancer drugs and more importantly maximizes the dose that can be deposited, which in turn improves the therapeutic efficacy. The carriers preferentially accumulate in tumor tissues by the enhanced permeability and retention (EPR) effect due to the characteristic leaky vasculature of tumors and insufficient lymphatic drainage. Apart from this so-called passive targeting, active targeting using carriers functionalized with tumor targeting ligands has been explored to enhance disease specificity and decrease systemic toxicity. However, because of the poor circulation kinetics and the rapid clearance of these carriers, tumor delivery is not markedly improved when actively targeting carriers are applied compared to passive accumulation from nontargeted carriers. As a result, the therapeutic index remains limited thus indicating the need for alternative strategies such as pretargeting. A pretargeting strategy using bsAbs and biotinylated liposomes loaded with technetium-99m-labeled DTPA improved targeting by four times, illustrating its possible use for selective delivery [128]. As a side note, the composition of liposomes can substantially influence the ability of biotinylated liposomes to bind SA [129].

One of the chemotherapeutic drugs identified by many researchers to substantially benefit from a pretargeted drug delivery system is doxorubicin (DOX). I.p. administration of SA decorated DOX-loaded liposomes resulted in a 3.7-fold increase in drug localization to the pretreated tumor site versus control liposomes [130]. The benefit of i.p. administration over i.v. injection for liposomal DOX carriers targeting i.p. inoculated tumors was also shown by Lehtinen et al. [131]. In the same study, the authors compared pretargeting vs. direct targeting vs. nontargeted liposomes, but this did not show any additional benefit of a pretargeting approach or targeting in general. For tumor necrosis treatment with DOX, however, pretargeting does seem to have potential. Here, treatment based on the biotinylated form of an antibody directed against degenerating cells located in tumor necrotic regions and SA modified liposomes as DOX carrier resulted in inhibited tumor growth within the first few days [132]. Khaw et al. tested a different approach to enhance tumor specificity by conjugating DOX to a polymer functionalized with DTPA to form a polymer prodrug conjugate that can bind to previously injected bsAbs directed against HER2 and DTPA [133]. Although the therapeutic efficacy was equivalent in HER2 positive human mammary carcinoma bearing mice pretargeted with a bsAb complex or treated with DOX only, the toxicity was significantly less as no total body weight loss was recorded for the pretargeted subgroup. This highlights the high target specificity of this method and its ability to increase the therapeutic index of DOX by overcoming the major limitation for its use: cardiotoxicity.

Tumor necrosis factor (TNF) receptor ligands were originally also recognized to have a strong antitumor potential, yet their poor tumor selectivity resulted in major side effects. A first attempt to increase the tumor concentration of TNF-alpha by means of pretargeting was done by Moro et al. in 1997 [134]. They pretreated cells in vitro with biotinylated antibodies followed by avidin to provide extra binding sites for the thereafter administered biotin-TNF prior to injection of those cells into mice. Consequently, mice bearing mouse mutant lymphoma xenografts appeared to be less tumorigenic, showing the potential of pretargeting to increase the binding and residence time of TNF on tumors. In vivo pretargeting with biotin-TNF increased the antitumor activity by at least five times without adding to the toxicity levels [135]. Tarrus et al. showed that a pretargeting approach with biotinylated recombinant TNF-related apoptosis-inducing ligands (TRAILs) targeting RGD-avidin, bound on angiogenic endothelial cells via the α_v_β_3_ integrin receptor, enhanced tumor cell death even further through increased tumor accumulation of these ligands [136].

For B-cell malignancies, apoptosis induction can be established by crosslinking CD20 at the surface of B-cells. A pretargeted nanotherapy using anti-CD20 antibody fragments conjugated to MORFs followed by the injection of polymer backbones decorated with multiple complementary MORFs significantly enhanced the pro-apoptotic activity compared to immunotherapy with the anti-CD20 antibody Rituximab alone [137]. Furthermore, this CD20 cross-linking amplification based on MORF pretargeting could be used to overcome the resistance to Rituximab [138]. Functionalized nanocarriers can also play a pivotal role in overcoming induced drug resistance. For example, patients resistant to the anti-HER2 monoclonal antibody trastuzumab could benefit from prelabeling their HER2 receptors with trastuzumab conjugated to a binding moiety for drug-loaded nanocarriers to still affect HER2 positive cells [139].

A delivery vehicle can additionally be utilized to carry high activity doses of radionuclides. To enable, for example, imaging of DOX-induced cardiotoxicity in small animals, nanocarrier pretargeting can be applied to localize a high specific radioactivity particularly at the target site [140]. Double labeling of nanocarriers such that the radioactivity load is distributed over the membrane and the inner aqueous phase, as was done by Rauscher et al., can further increase the deposited doses [141]. Liposomes also offer the opportunity to transport Auger-electron-emitting radionuclides into the cell nucleus to get them in close vicinity to DNA where they are cytotoxic. Using a HER2-directed pretargeting approach, a 70% survival was reached demonstrating the potential of pretargeting with Auger-electrons [142].

In some cases, passive NP targeting still has its advantages. Delineation of tumor margins with positron emission tomography can be established as a consequence of the accumulation of radiopharmaceutical liposomes via the EPR effect. However, due to long circulation time of liposomes, long-lived radionuclides like zirconium-89 are necessary. Therefore, Brand et al. designed the so-called on-demand bioorthogonal removal of circulating TCO-tagged liposomal NPs to decrease exposure to nontargeted tissues and allow imaging at an earlier time point [143]. Most recently, pretargeting has extended its application to the field of liver radioembolization. In this case, pretargeting is used to better correlate the scout scan (a scan that is used to predict the intra- and extrahepatic distribution of activity) to the therapeutic dose. Here, the strong interaction between cyclodextrin and adamantine was employed; an alternative pretargeting method [144,145].

**Figure 4:**
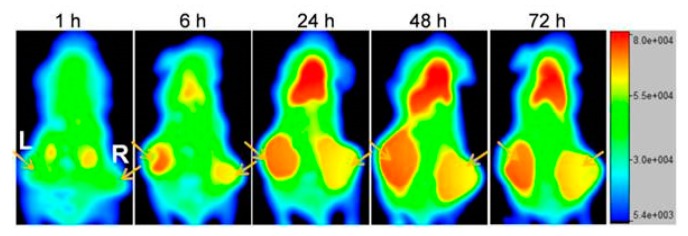
Amplification of the nanoconjugates (NCs) tumor targeting performance via metabolic engineering of tumor cells. Whole-body fluorescence imaging of mice bearing human CRC tumors left and right at 1, 6, 24, 48, and 72h after injection of the azido-/Cy5-NCs. The right tumor was injected with PBS as control and the left tumor was injected with Ac_4_ManDBCO daily for three days prior to injection of the NC to label the cancer cells. The arrows point at the tumors. This research was originally published in Theranostics. Wang et al. In vivo targeting of metabolically labeled cancers with ultra-small silica nanoconjugates. *Theranostics* **2016**, *6*, 1467–1476. [149]

In recent years, there has been an increasing interest in introducing artificial receptors on tumors by means of metabolic glycoengineering. This technique involves cell treatment with metabolic precursors in the form of unnatural monosaccharides containing azido groups. These azido groups are inserted into the surface glycan via the intrinsic metabolic pathway of the cell, making azido groups available for dibenzocyclooctyne (DBCO) to bind. Combining bioorthogonal chemistry (i.e., the copper-free click chemistry reaction between an azido group and DBCO) with this cell surface labeling strategy enables cell targeting by functionalized NPs with high efficiency. Pretargeting via metabolic glycoengineering is shown to be dose-dependent, meaning that binding of the functionalized NP can be controlled by changing the concentration of the metabolic precursor injected into the tumor [146]. Layek et al. provided evidence of the tumor specificity of this method and illustrated its potential to significantly inhibit tumor growth and improve survival in an orthotopic metastatic ovarian tumor model when administering glycoengineered mesenchymal stem cells and paclitaxel-loaded functionalized NPs [147]. The artificial chemical receptors presenting azido groups for bioorthogonal click chemistry can also be generated in vivo by enveloping the metabolic precursors in NPs and letting the localization in targeted tumor tissue be guided by the EPR effect [148]. The feasibility of a reversed approach by generating large-sized DBCO groups on tumor cells in contrast to the previously used small-sized azido groups for cell labeling was demonstrated by Wang et al. [149]. The authors also clearly illustrated the benefit of pretargeting via metabolic engineering over passive targeting through the EPR effect for tumor accumulation of nanoconjugates (Figure 4). Introducing receptor-like Tz groups has also been shown to significantly enhance NP tumor targeting [150]. Artificially installing receptors is a way to overcome tumor heterogeneity and the limitation of low numbers of biological receptors available for targeted therapies. Alternatively, Yang et al. considered a dual targeting approach to deal with tumor subpopulations using a cocktail of anti-CD20 and anti-TAG-72 FPs to attract biotinylated NPs [151].

## 6. Conclusions and Perspectives

Pretargeting has been shown to be a feasible and a promising strategy for therapeutic purposes. PRIT has proven to be superior over RIT in terms of more favorable T:B ratios, reduced toxicity, and improved efficacy. The low toxicity profile of PRIT allowed for treatment intensification, as higher maximum tolerated doses could be safely administered, and was therefore particularly suited to be part of a combination therapy regimen. As a result of the impressive results obtained with PRIT, the use of pretargeting has expanded to alpha-particle therapy and drug delivery systems. The scope of pretargeting will grow further now that internalizing targets have presented themselves as potential targets. The success of treatment is largely dependent on tumor antigen expression; hence, imaging beforehand could assist, encouraging the application of pretargeting strategies for theranostic applications. The importance of choosing a suitable target was further underlined by the cross-reactivity with off-target tissues observed for e.g., EpCAM-directed PRIT. The technique to introduce artificial receptors on cell surfaces provides an alternative when target cells lack appropriate molecular biomarkers. Furthermore, pretargeting with smaller targeting constructs has shown less toxicity due to faster clearance and better binding, thereby decreasing the risk of nephrotoxicity normally seen for small fragments. The future will have to show whether this will drive the development of smaller targeting compounds such as peptides for pretargeted therapy as well.

Despite its immense promise, pretargeted strategies have not yet been implemented in the clinic. This is mostly due to the lack of an appropriate bioconjugation technology. Over the years, different pretargeting techniques have emerged, and each proved to have advantages and disadvantages. Overall, the avidin–biotin system has been studied most frequently in both preclinical and clinical studies. An important advantage of this method is the high binding affinity of biotin for SA. In addition, SA has four binding sites for biotin, which, together with the high binding affinity, results in high and persistent tumor uptake. However, this system is hampered by drawbacks in the form of immunogenicity evoked by SA, and the in vivo stability of biotin which is dependent on biotinidase activity and the competition with endogenous biotin. Apart from the avidin–biotin system, a lot of clinical research has been performed with bispecific antibodies, mostly directed at CEA. These studies marked the need for fully humanized constructs to avoid a significant human anti-mouse antibody response induced by murine and chimeric constructs. In this case, the complex production and costly process is a major disadvantage hampering clinical translation. In addition, as a consequence of the reversibility of the bispecific antibody–hapten interaction, a generally lower tumor uptake is observed with this pretargeting method compared to the other methods.

The newer pretargeting methods based on complementary oligonucleotides and bioorthogonal click chemistry might offer solutions for the primary drawback of the avidin–biotin system and the use of bispecific antibodies, because these methods do not interfere with biological processes and therefore evade immunogenicity. A potential disadvantage of using complementary oligonucleotides for pretargeting is the limited in vivo stability of these constructs. However, PRIT using this emerging strategy is currently only studied at the preclinical stage and methods are being developed to overcome this limitation. When it comes to bioorthogonal click chemistry, a major advantage of this method is that the reactions are selective, fast, and efficient. To date, clinical studies using this method have not been performed, but results of preclinical studies are promising.

The challenge of pretargeting being a multistep process makes it more difficult and costly to develop, but the fact that PRIT can provide a treatment option for chemoresistant or end-stage patients illustrates that it is worthwhile to improve the pretargeting technology. Moving forward, without doubt, new strategies will be explored that will in turn stimulate the use of pretargeting in novel ways.
